# Investigating the Relationship Between Problematic TikTok Use, Eating Disorder Symptoms, Psychological Symptoms, and Metacognition Among University Students

**DOI:** 10.1192/j.eurpsy.2026.11368

**Published:** 2026-07-31

**Authors:** F. Obuća, O. Aydın, P. Ünal-Aydın

**Affiliations:** Psychology, International University of Sarajevo, Sarajevo, Bosnia and Herzegovina

## Abstract

**Introduction:**

The rise of social media platforms, particularly TikTok, has raised concerns regarding their potential impact on mental health and well-being. Excessive or problematic use of TikTok may be associated with maladaptive outcomes such as disordered eating behaviors, heightened psychological distress, and dysfunctional metacognitive processes. Despite its global popularity, research on TikTok use and its psychological correlates among young adults in Bosnia and the wider region remains limited, making this study an important contribution to the field.

**Objectives:**

This study aimed to examine the relationship between eating disorder symptoms, as measured by the Eating Disorder Examination–Questionnaire Short (EDE-QS), and predictors including problematic TikTok use, metacognitive beliefs, and symptoms of depression, anxiety, and stress in a sample of university students.

**Methods:**

A total of 685 university students participated in this study through convenience sampling. Data were collected using the Sociodemographic Data Form (age, gender, nationality, faculty, average daily screen time, average daily TikTok use, and checking TikTok first thing in the morning), TikTok Addiction Scale (TTAS), Eating Disorder Examination–Questionnaire Short (EDE-QS), Metacognition Questionnaire-30 (MCQ-30), and Depression Anxiety Stress Scales (DASS-21). Descriptive statistics, correlation analyses, and multiple linear regression were conducted to evaluate associations between variables.

**Results:**

Regression analysis revealed that higher levels of stress, TikTok addiction withdrawal symptoms, positive metacognitive beliefs about worry, and the perceived need to control thoughts were significant positive predictors of disordered eating symptoms as measured by the EDE-QS.

**Conclusions:**

The findings highlight the complex interaction between problematic TikTok use, maladaptive metacognitions, and psychological distress in predicting disordered eating symptoms among young adults. These results underscore the importance of addressing social media behaviors and cognitive-emotional processes in preventive and clinical interventions targeting eating disorders in student populations.

**Disclosure of Interest:**

None Declared

